# Concurrent Catastrophic Antiphospholipid Syndrome, Hemophagocytic Lymphohistiocytosis, and Myocarditis Leading to Multi-organ Failure in a Patient With Systemic Lupus Erythematosus and Antiphospholipid Syndrome

**DOI:** 10.7759/cureus.116801

**Published:** 2026-09-23

**Authors:** Shiva Chauhan, Maymuna Suleman, Antoni Chan

**Affiliations:** 1 Internal Medicine, Royal Berkshire Hospital, Reading, GBR; 2 Rheumatology, Royal Berkshire Hospital, Reading, GBR; 3 Clinical Medicine, University of Reading, Reading, GBR

**Keywords:** antiphospholipid syndrome (apls), autoimmune overlap, catastrophic antiphospholipid syndrome (caps), chronic immunosuppression, hemophagocytic lymphohistiocytosis (hlh), lupus myocarditis, macrophage activation syndrome (mas), multi-organ failure, refractory disease, systemic lupus erythematosus (sle)

## Abstract

Catastrophic antiphospholipid syndrome (CAPS) is a fulminant and life-threatening manifestation of antiphospholipid syndrome (APLS), often presenting with rapid multi-organ dysfunction and high mortality despite aggressive therapy. Its associations with hemophagocytic lymphohistiocytosis (HLH) and lupus myocarditis are not well understood.

We report the case of a 43-year-old woman with systemic lupus erythematosus (SLE) and secondary APLS who developed CAPS following elective mitral valve repair. Her course was complicated by renal cortical necrosis, recurrent lupus myocarditis, refractory immune thrombocytopenia, sepsis, and HLH, requiring multiple immunosuppressive modalities including corticosteroids, intravenous immunoglobulin, plasma exchange, rituximab, cyclophosphamide, and anakinra. Despite intensive multidisciplinary management across rheumatology, cardiology, nephrology, hematology, infectious diseases, and intensive care, she suffered progressive multi-organ failure and transitioned to palliative care.

This case highlights the diagnostic and therapeutic complexity of overlapping autoimmune syndromes, the risks of surgical triggers in predisposed patients, and the challenges of balancing immunosuppression against infection in the setting of CAPS. It underscores the need for dynamic multidisciplinary collaboration, early recognition of HLH and lupus myocarditis overlap, and timely integration of palliative care when maximal therapy fails.

## Introduction

Catastrophic antiphospholipid syndrome (CAPS) is a rare, life-threatening variant of antiphospholipid syndrome (APLS), characterized by widespread small-vessel thromboses resulting in multi-organ failure. CAPS occurs in less than 1% of patients with APLS [1] and is often associated with systemic lupus erythematosus (SLE) [1-3]. Clinical manifestations typically arise from either thrombotic events or systemic inflammation [1-3]. The kidneys are most frequently involved (73%), followed by the lungs (58.9%), central nervous system (55.9%), and heart (49.7%) [2]. Hemophagocytic lymphohistiocytosis (HLH) is a severe hyperinflammatory syndrome that may complicate SLE, while lupus myocarditis is an inflammatory cardiac manifestation of SLE. Both are relevant to this case because their clinical features may overlap with CAPS and severe systemic inflammation, creating substantial diagnostic and therapeutic uncertainty.

The diagnosis of CAPS requires evidence of small-vessel thrombosis involving at least three organ systems, with manifestations developing rapidly within a week, alongside the persistent presence of antiphospholipid antibodies and histopathological evidence of small-vessel occlusions [1-3]. Diagnosis is complicated by significant heterogeneity in clinical presentation [2,3], non-specific serological testing [1], and difficulty in histological confirmation, especially in acute settings [1,2]. Moreover, infection or inflammation can lead to false-positive antibody results, while thrombotic events can lead to false-negative results, which can further confound diagnosis [1,2].

Treatment typically involves a multi-pronged approach [3]. The first focus should be to remove the triggering event. The second focus should be for therapeutic anticoagulation with a target international normalized ratio (INR) > 2.0 to prevent thrombotic events [1]. A third focus should be to blunt complement and cytokine activation with immunomodulators [2]. CAPS has been typically associated with a mortality rate as high as 50%. However, between 2001 and 2006, this mortality level fell from 53% to 33% due to earlier recognition with aggressive anticoagulation and immunosuppression given at an earlier stage [1,2]. Mortality is typically due to cerebral involvement (27.2%), followed by cardiac events (19.8%) and infections (19.8%) [4]. Additionally, SLE has been suggested to be the only prognostic factor implicated in higher mortality [4].

We present a rare and complex case of a patient with longstanding SLE and secondary APLS who developed catastrophic multi-organ failure following elective mitral valve repair. Her course was marked by recurrent admissions, multi-system involvement, recurrent lupus myocarditis, HLH and eventual transition to palliative care - illustrating the devastating potential of CAPS despite intensive therapy.

## Case presentation

Initial presentation and background

A 43-year-old woman with SLE, diagnosed in 2002, and secondary APLS was admitted multiple times between January and May 2025 with progressive multi-system deterioration requiring extensive multidisciplinary input. Her past medical history included recurrent venous thromboembolism, multiple miscarriages, and two ischemic strokes in 2011 and 2012. She had been maintained on long-term immunosuppression with mycophenolate mofetil 1500 mg twice daily, hydroxychloroquine 400 mg once daily, and prednisolone 5 mg once daily.

Throughout 2024 she experienced recurrent chest pain and breathlessness and was found to have severe mitral regurgitation secondary to lupus myocarditis. She was therefore listed for elective mitral valve repair to improve cardiac function and quality of life, with the aim of reducing chronic myocardial inflammation. Preoperative rheumatology assessment demonstrated clinically and serologically quiescent SLE, with an SLE Disease Activity Index 2000 (SLEDAI-2K) score of 0, anti-double-stranded DNA (anti-dsDNA) of 2, normal complement C3 and C4 levels, erythrocyte sedimentation rate (ESR) of 13 mm/h, C-reactive protein (CRP) of 1.4 mg/L, platelet count of 140 × 10⁹/L, and hemoglobin of 112 g/L. Multidisciplinary review identified no requirement for additional SLE-directed treatment before surgery, and her existing mycophenolate mofetil, hydroxychloroquine, and low-dose prednisolone were continued perioperatively. Preoperative antiphospholipid antibody titers were not obtained; therefore, a sequential perioperative rise in antiphospholipid antibody levels could not be assessed.

**Figure 1:**
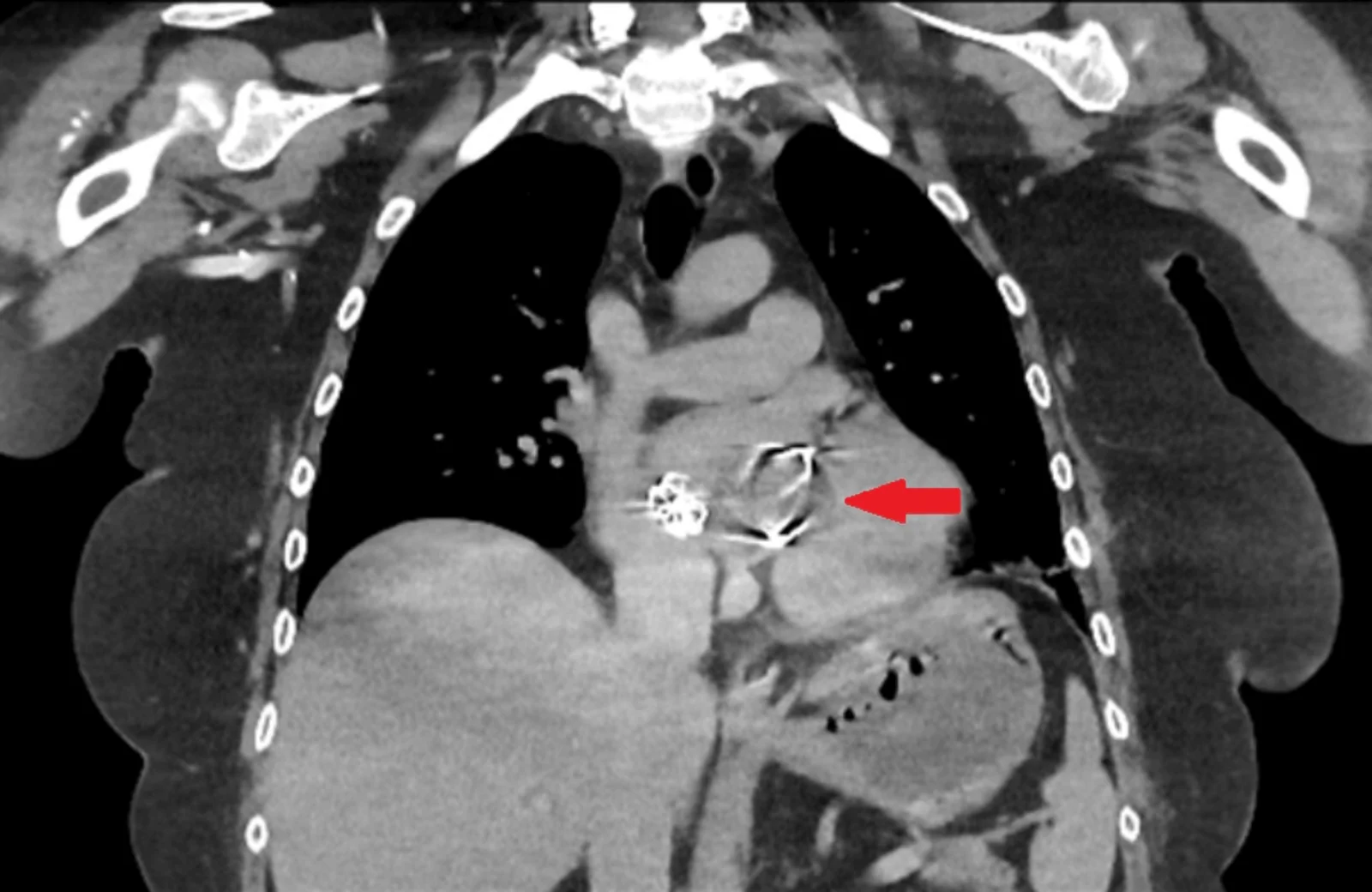
Coronal contrast-enhanced computed tomography (CT) image of the thorax demonstrating metallic artifact from the mitral valve repair (red arrow).

She underwent elective mitral valve repair in January 2025 (Figure 1). The immediate postoperative period was complicated by immune thrombocytopenia (ITP) and multi-territorial embolic infarcts involving the left frontal lobe and frontal operculum. The overall clinical course, including the major complications, interventions, and outcomes across the three admissions, is summarized in Table 1. 

**Table 1:** Timeline of clinical events, major interventions, complications, and outcomes. AKI: acute kidney injury; ALT: alanine aminotransferase; APLS: antiphospholipid syndrome; CAPS: catastrophic antiphospholipid syndrome; CMR: cardiac magnetic resonance imaging; CMV: cytomegalovirus; CPAP: continuous positive airway pressure; HLH: hemophagocytic lymphohistiocytosis; ICU: intensive care unit; IVIG: intravenous immunoglobulin; SLE: systemic lupus erythematosus.

Period	Clinical phase	Key findings and complications	Major interventions	Outcome or decision
Before January 2025	Background SLE and secondary APLS	SLE diagnosed in 2002; recurrent venous thromboembolism, multiple miscarriages, and ischemic strokes in 2011 and 2012. Severe mitral regurgitation was attributed to lupus myocarditis	Long-term mycophenolate mofetil, hydroxychloroquine, and prednisolone; elective mitral valve repair planned	Surgery undertaken to improve cardiac function and quality of life
January 2025	Mitral valve repair and early postoperative deterioration	Postoperative immune thrombocytopenia and multi-territorial embolic infarcts involving the left frontal lobe and frontal operculum	Postoperative multidisciplinary management	Rapid thrombotic and hematologic deterioration followed surgery
January-March 2025	Post-surgical probable CAPS	Within one week of surgery, the patient developed bilateral renal cortical necrosis, splenic infarction, thrombocytopenia, active urinary sediment, and acute renal failure. Lupus anticoagulant and anticardiolipin antibodies were positive	ICU admission, renal replacement therapy, high-dose corticosteroids, mycophenolate mofetil, IVIG, rituximab, and five plasma-exchange sessions. Romiplostim was used for persistent thrombocytopenia	Renal function initially improved, but thrombocytopenia and recurrent Pseudomonas sternotomy-wound infection persisted
March-April 2025	Recurrent lupus myocarditis and further immunosuppression	Chest pain, breathlessness, hemoptysis, troponin 3754 ng/L, and CMR findings supportive of myocarditis. Fluid overload, worsening thrombocytopenia, pneumonia, recurrent renal dysfunction, and low-level CMV viremia subsequently developed	Three intravenous methylprednisolone pulses reduced troponin to 1834 ng/L; further IVIG, Euro-Lupus cyclophosphamide, oral steroid taper, CPAP, antimicrobial therapy, romiplostim, and further dialysis	Temporary clinical improvement permitted discharge with rheumatology, hematology, and renal follow-up
April-May 2025	Severe infection and probable HLH overlap	Pseudomonas right-leg cellulitis, fever, cytopenias, oligo-anuric AKI, marked ferritin and ALT elevation, progressive fluid overload, and delirium. HScore was 175, corresponding to an estimated 54-70% probability of HLH	Broad-spectrum antimicrobial therapy, anakinra 400 mg on alternate days, dexamethasone, renal replacement therapy, and supportive management	Laboratory indices initially improved but subsequently deteriorated; anakinra was discontinued
Early May 2025	Progressive multi-organ failure and therapeutic exhaustion	Rising inflammatory markers, recurrent infection, worsening organ dysfunction, and no response to a final IVIG trial despite multiple immunomodulatory treatments	Multidisciplinary review involving rheumatology, nephrology, hematology, cardiology, intensive care, infectious diseases, and palliative care	Further immunosuppression was judged unlikely to provide benefit. Treatment transitioned to comfort-focused care, and the patient died approximately four months after surgery

First admission: post-surgical CAPS with renal failure (January 2025 to March 2025)

**Figure 2:**
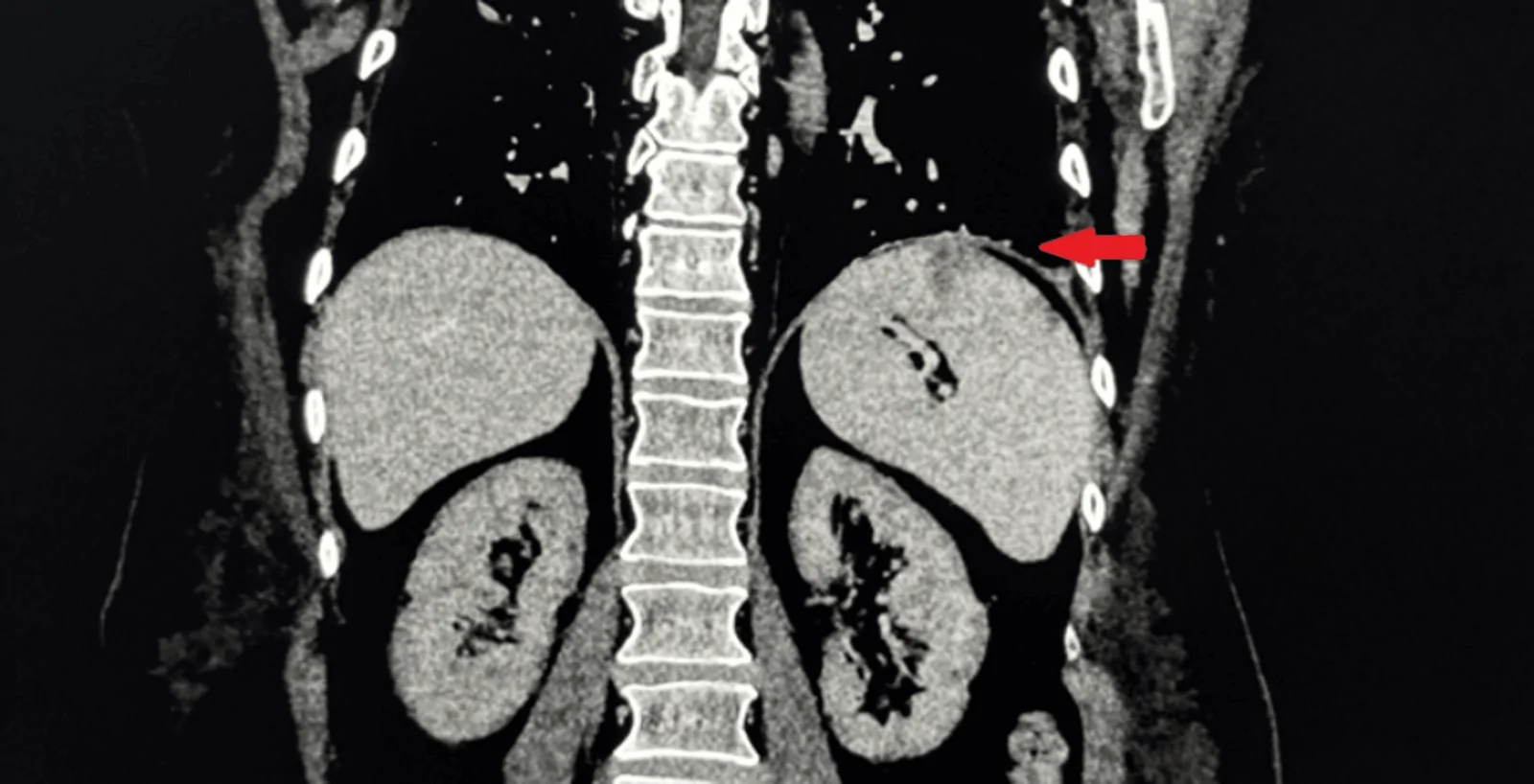
Coronal contrast-enhanced CT abdomen demonstrating a wedge-shaped hypodense region in the spleen indicating splenic infarct (red arrow), consistent with thrombotic involvement in catastrophic antiphospholipid syndrome (CAPS).

Post-operatively, she developed CAPS with bilateral renal cortical necrosis, likely triggered by surgery. Urinalysis revealed active urinary sediment with both blood and protein. She developed thrombocytopenia as well as splenic infarcts (Figure 2). Manifestations had rapid onset, occurring within a week. She was positive for anticardiolipin and lupus anticoagulant antibodies. Using the preliminary international CAPS classification criteria [1], she demonstrated involvement of at least three organs, including multi-territorial cerebral infarcts, bilateral renal cortical necrosis, and splenic infarction. These manifestations developed within one week of mitral valve repair, and laboratory evidence included positive lupus anticoagulant and anticardiolipin antibodies in the context of established APLS. Histopathological confirmation of small-vessel occlusion was not obtained; therefore, the presentation fulfilled the CAPS international classification criteria for probable rather than definite CAPS [1].

This episode of CAPS precipitated acute renal failure necessitating intensive care unit (ICU) admission and renal replacement therapy. Renal function improved following plasma exchange, and hydroxychloroquine was discontinued due to toxicity concerns in severe renal failure.

Her postoperative recovery was further complicated by sternotomy wound breakdown with recurrent *Pseudomonas* infection. This was managed with meropenem then aztreonam and vacuum-assisted closure (VAC) dressings, a form of negative-pressure therapy that promotes wound healing by removing exudate and stimulating granulation tissue. Recurrent severe thrombocytopenia required weekly romiplostim infusions to support continued management of thrombotic risk. Recurrent thrombocytopenia was often refractory, necessitating eltrombopag and complicated therapeutic anticoagulation, as platelet counts frequently fell below 50 × 10⁹/L. Enoxaparin dosing was individualized under hematology guidance using anti-factor Xa levels and adjusted according to the patient’s hematological and clinical status.

Second admission: recurrent lupus myocarditis and immunosuppression (March 2025 to April 2025)

She re-presented with chest pain, breathlessness, and hemoptysis. Troponin was > 3000 ng/L, raising concern for recurrent lupus myocarditis and an SLE/APLS flare. A contemporaneous ECG on 19 March 2025 demonstrated lateral T-wave inversion with normal sinus rhythm at approximately 83 beats/min. Repeat ECG on 23 March showed reduction in the depth of these T-wave inversions, with subsequent ECGs by July 2025 demonstrating near-complete resolution of the repolarization abnormalities. These dynamic repolarization changes were considered supportive of an acute myocardial process in the context of the marked troponin elevation and other clinical and cardiac magnetic resonance imaging (CMR) findings.

**Figure 3:**
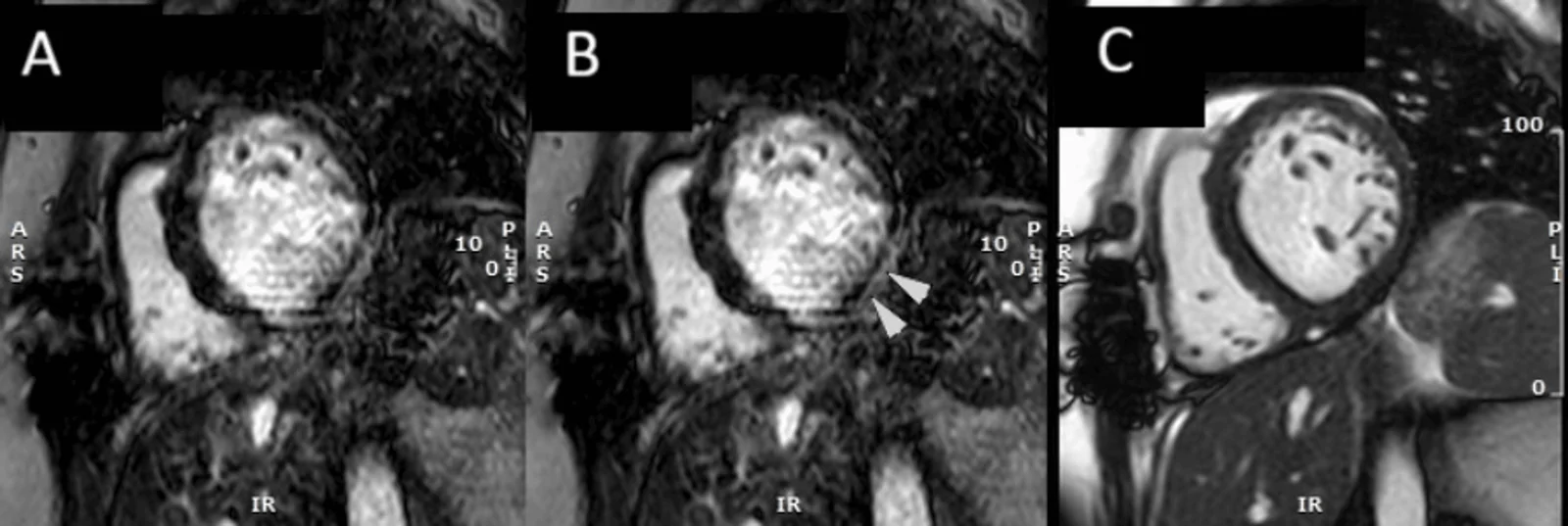
Short-axis cardiac MRI. (A-B) Delayed gadolinium enhancement images demonstrating mid-wall/subepicardial enhancement (arrows) supportive of nonischaemic myocardial injury consistent with myocarditis. (C) Matching short-axis cine image, shown to confirm this signal represents true late gadolinium enhancement rather than subepicardial fat. Image artifact is present due to difficulty with breath-holding.

CMR demonstrated anterolateral and inferolateral mid-wall/subepicardial late gadolinium enhancement in a nonischemic pattern, with a left ventricular ejection fraction of approximately 50% (Figure 3). Comprehensive tissue characterization was limited because T1 and T2 mapping were unavailable on the scanner used, while the acquired T2-weighted images were nondiagnostic because of breathlessness, tachycardia, difficulty with breath-holding, respiratory motion, and ECG-gating artifact. As late gadolinium enhancement alone could not reliably distinguish active from previous myocarditis, recurrent lupus myocarditis was considered the most likely diagnosis through integration of the clinical presentation, marked troponin elevation, available CMR findings, recent unobstructed coronary arteries, and subsequent biochemical response to intravenous methylprednisolone. Troponin levels fell from 3754 ng/L to 1834 ng/L after three pulses of intravenous methylprednisolone.

This interpretation was consistent with her previous myocarditis episode in May 2024, which had been associated with low complement levels and had responded favorably to cyclophosphamide, supporting an autoimmune etiology. During the current admission, the treating teams likewise favored lupus myocarditis, and no alternative infectious etiology was documented as a contemporaneous concern. Histopathological confirmation was not obtained.

A further five-day course of intravenous immunoglobulin (IVIG) was commenced due to worsening thrombocytopenia and renal function, followed by cyclophosphamide under the EuroLupus protocol with tapering oral steroids.

**Figure 4:**
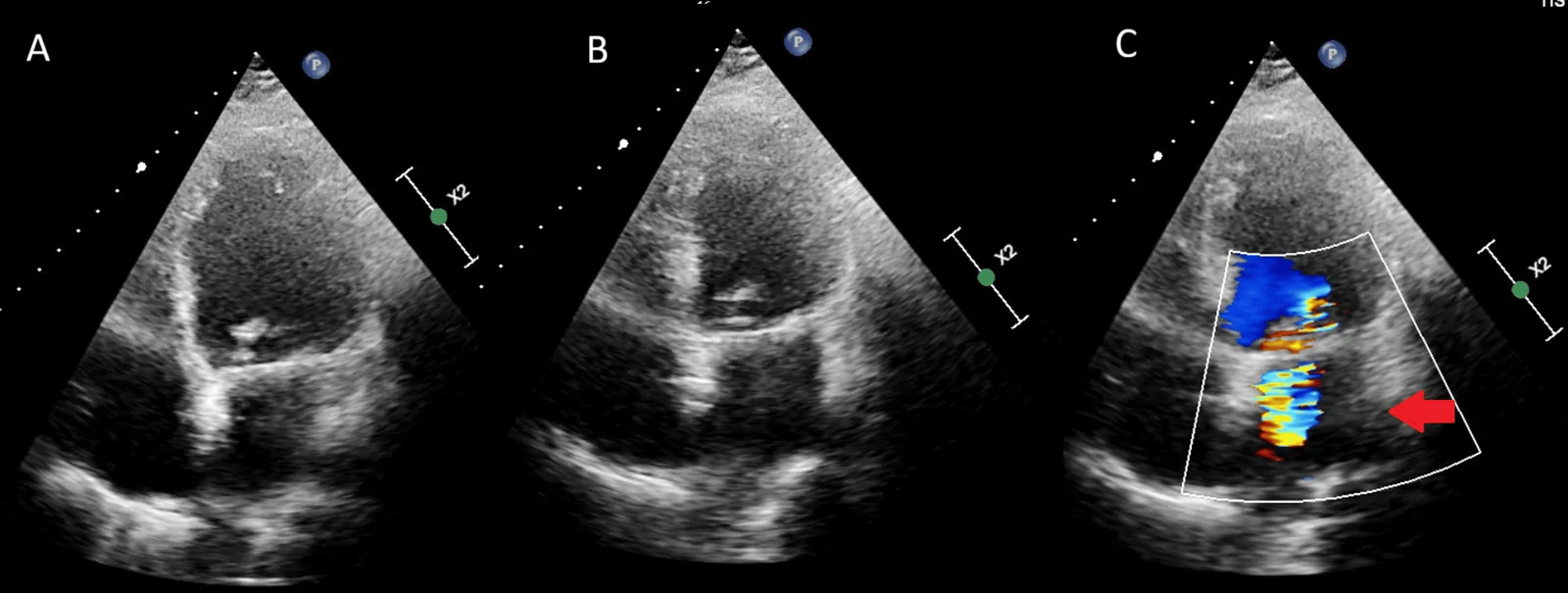
Representative still frames from transthoracic echocardiography. Global left ventricular contractility was assessed using the complete dynamic cine study, which demonstrated mild systolic impairment with a left ventricular ejection fraction of 45–50%. (A–B) Representative grayscale echocardiographic views from the study. (C) Color Doppler demonstrating moderate residual mitral regurgitation, with the regurgitant jet directed into the left atrium (arrow).

**Figure 5:**
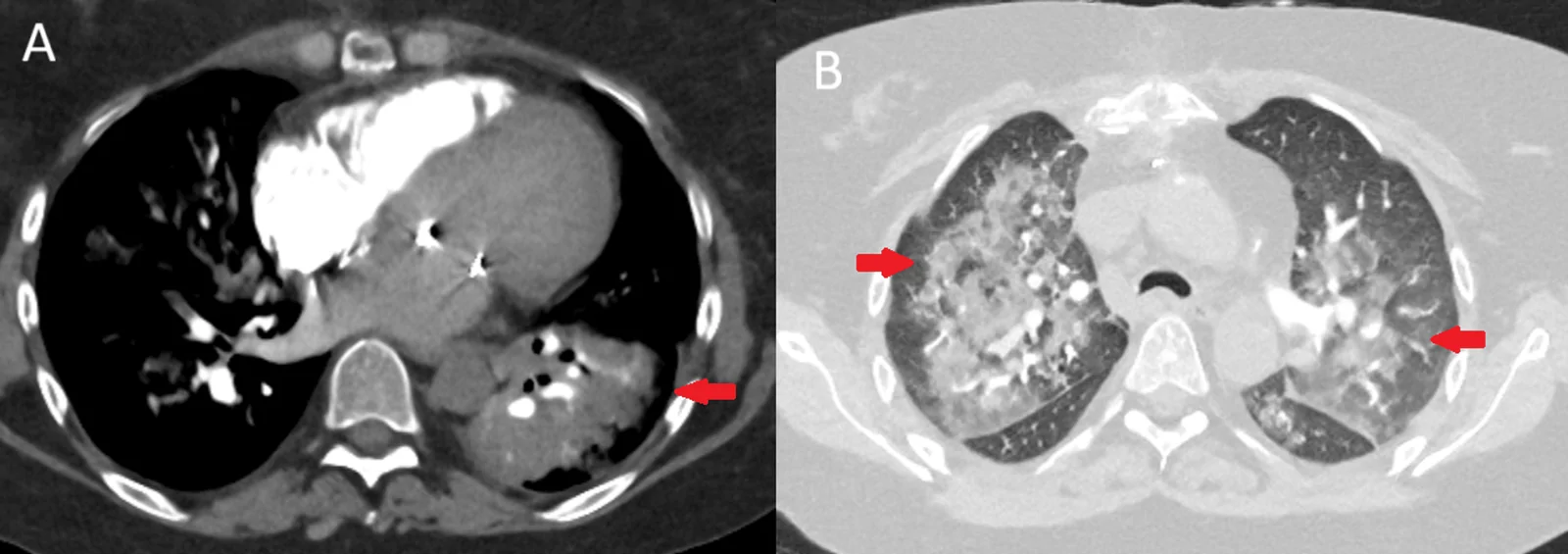
CT pulmonary angiography. (A) Mediastinal window demonstrating a large dense consolidation in the left lower lobe (arrow), with additional patchy opacities in right lung. (B) Lung window confirming extensive left lower lobe consolidation with extensive bilateral ground-glass opacities (arrows), consistent with severe hospital-acquired pneumonia. No pulmonary embolus was identified.

She developed multifactorial fluid overload requiring intravenous diuresis and continuous positive airway pressure (CPAP) support. Echocardiography suggested mild left-ventricular impairment (45-50%) on dynamic cine assessment and moderate residual mitral regurgitation (Figure 4); invasive angiography was deferred given recent unobstructed coronaries. Persistent hypertension with diastolic pressures > 100 mmHg required escalation of amlodipine to 10 mg daily. Thrombocytopenia worsened, necessitating repeated romiplostim, and hospital-acquired pneumonia with bilateral consolidation on CT pulmonary angiography (CTPA) led to another ICU admission (Figure 5). Labile renal function required further dialysis, while multifactorial iron-deficiency anemia was treated with intravenous iron and transfusions. A low positive cytomegalovirus (CMV) titer (92 IU/mL) prompted valganciclovir therapy. After gradual improvement she was discharged with close rheumatology, hematology, and renal follow-up.

Third admission: refractory cellulitis, HLH overlap, and multi-organ failure (April 2025 to May 2025)

In April 2025, she was readmitted after routine surveillance identified right-leg cellulitis with fever, raising concern for neutropenic sepsis and possible necrotizing fasciitis. Examination revealed tracking cellulitis extending to the medial thigh with hemorrhagic and tense bullae over the calf. Wound cultures grew Pseudomonas, and she required multiple antibiotics, including clindamycin, co-trimoxazole, teicoplanin, gentamicin, meropenem, fluconazole, piperacillin-tazobactam, caspofungin, linezolid, vancomycin, and aciclovir. Surgical exploration by orthopedics excluded necrotizing fasciitis.

She developed oligo-anuric acute kidney injury (AKI) on chronic kidney disease (CKD) requiring hemodialysis, which was discontinued once urine output recovered. Thrombocytopenia persisted despite romiplostim and eltrombopag, with only marginal platelet improvement, and a petechial pruritic rash appeared on the arms, abdomen, and eyelids.

Grossly elevated ferritin and alanine aminotransferase (ALT) raised concern for HLH, prompting treatment with anakinra 400 mg subcutaneously on alternate days. Laboratory indices initially improved but soon worsened. HLH was assessed using the HScore, an adult probability model for reactive hemophagocytic syndrome. Her HScore was 175, corresponding to an estimated 54-70% probability of HLH. In the context of fever, persistent cytopenias, marked hyperferritinemia, transaminitis, and progressive organ dysfunction, this supported a working diagnosis of probable HLH overlap. The differential diagnosis included severe infection with sepsis-associated coagulopathy, active SLE, ischemic hepatic injury, and treatment-related organ dysfunction. Sepsis-related disseminated intravascular coagulation could not be definitively excluded from the available findings. However, taken together, the clinical and biochemical features described above favored probable HLH rather than infection or hepatic ischemia alone. The initial biochemical improvement following anakinra provided additional supportive, although not definitive, evidence of a hyperinflammatory component. Furthermore, she developed progressive fluid overload, fluctuating delirium, poor glycemic control requiring insulin adjustment, and oral thrush with mucositis treated with fluconazole. She was commenced on dexamethasone 6 mg subcutaneously daily while anakinra was later discontinued.

Across the successive admissions, treatment escalation was guided by the emergence of organ-threatening manifestations and response to preceding therapies. Intravenous methylprednisolone was administered for suspected recurrent lupus myocarditis with marked troponin elevation; IVIG was added for worsening thrombocytopenia and renal function; cyclophosphamide was subsequently commenced for severe recurrent autoimmune disease; and anakinra was introduced when the clinical and biochemical findings raised concern for probable HLH. Despite transient responses, the patient developed recurrent infection, rising inflammatory markers, and progressive renal, hematological, neurological, and cardiopulmonary dysfunction.

By early May 2025, her inflammatory markers continued to rise despite maximal therapy, and a final trial of IVIG produced no clinical or biochemical response. Following multidisciplinary review involving rheumatology, nephrology, hematology, cardiology, intensive care, infectious diseases, and palliative care, further immunosuppression was considered unlikely to provide meaningful benefit and increasingly likely to worsen infection and treatment toxicity. No single prognostic scoring system determined the transition to palliative care; the decision was based on the longitudinal clinical trajectory. The combination of progressive multi-organ failure, failure of multiple immunomodulatory therapies, recurrent infection, and continued clinical deterioration therefore prompted transition to comfort-focused care. She passed away peacefully after medications were rationalized for symptom control. Her CAPS manifestations began within one week of mitral valve repair, and progressive multi-organ deterioration culminated in death in early May 2025, approximately four months after surgery.

Written informed consent for publication of this case was obtained from the patient's next of kin.

## Discussion

This case highlights several important themes, including the role of surgical triggers, the refractory and recurrent nature of CAPS, the dilemmas of immunotherapy stacking, a timely escalation to palliative care, and the complex association between CAPS, SLE, HLH, and lupus myocarditis.

Surgical triggers: mitral valve repair as a precipitant of CAPS

Recognized precipitating factors for CAPS include infection, pregnancy, malignancy, surgery and anticoagulation withdrawal [1,2]. Approximately 60% of patients have at least one identifiable trigger; often these factors can exist concomitantly [1]. This case illustrates a rare presentation of post-surgical CAPS in a patient with longstanding SLE and secondary APLS. Uniquely, her trigger was an elective mitral valve repair due to symptomatic lupus myocarditis. A retrospective case series study of 18 patients identified six patients where surgery was a trigger; sepsis (18 patients) and cancer (18 patients) were more consistent overarching triggers [5]. This suggests that, in predisposed individuals with heightened complement or cytokine responses, surgical intervention could trigger further inflammatory cascades, a so-called ‘double-hit’ response [1,2].

A pediatric case report, describing an 11-year-old who developed CAPS following percutaneous cardiac catheterization for aortic re-coarctation, discusses the paucity of literature exploring the relationship between cardiac surgery and CAPS development [6]. Another case report examines CAPS registry data; it is noted that 35 of 282 patients had surgery as a trigger with 31.4% (11/35) cases involving gynecological surgery and 25.7% (9/35) involving gastroenterology surgery; whereas cardiac surgery was never cited as a precipitant [7]. This suggests cardiac surgery is a very rare precipitant of CAPS.

Overlapping syndromes: SLE, CAPS and HLH

A key feature in this case was diagnostic overlap. Many clinical signs - renal dysfunction, cytopenia, neurologic events - are shared between CAPS, SLE flares and HLH/MAS. The association between SLE and APLS is well documented [1-3]. Clinical and immunological overlap between SLE and APLS can make distinction challenging, and misclassification between the two remains a recognized concern [1]. The overlapping and distinguishing clinical features of CAPS, HLH, and active SLE, together with their interpretation in this patient, are summarized in Table 2.

**Table 2:** Overlapping and distinguishing features of CAPS, HLH, and SLE activity. ALT: alanine aminotransferase; APLS: antiphospholipid syndrome; CAPS: catastrophic antiphospholipid syndrome; HLH: hemophagocytic lymphohistiocytosis; IVIG: intravenous immunoglobulin; SLE: systemic lupus erythematosus.

Feature	CAPS [1-3,8,9]	HLH [10-12]	SLE activity/lupus myocarditis [8,10]	Interpretation in this patient
Predominant pathological process	Widespread small-vessel thrombosis associated with antiphospholipid antibodies, complement activation, and systemic inflammation	Severe hyperinflammatory syndrome associated with excessive immune activation	Autoimmune inflammation affecting one or more organ systems	Thrombotic, inflammatory, infectious, and cardiac processes occurred sequentially and overlapped
Typical clinical tempo	Rapid development of multi-organ involvement, usually within one week	Progressive systemic inflammation with clinical and biochemical deterioration	Variable; may present as an acute flare or recurrent organ-specific disease	CAPS manifestations developed within one week of mitral valve repair, while probable HLH emerged later during infection and prolonged immunosuppression
Principal clinical features	Multi-organ thrombosis, ischemic injury, renal dysfunction, neurological events, and thrombocytopenia	Fever, cytopenias, liver dysfunction, hyperferritinemia, and multi-organ dysfunction	Organ-specific inflammation, cytopenias, renal dysfunction, neurological manifestations, or myocarditis	The patient developed cerebral, renal, and splenic ischemic injury, followed by recurrent myocarditis, infection, cytopenias, and hyperinflammation
Thrombotic manifestations	Central diagnostic feature	Not a defining feature, although thrombosis may coexist	Not a defining feature of SLE activity unless associated with secondary APLS	Multi-territorial cerebral infarcts, bilateral renal cortical necrosis, and splenic infarction strongly supported CAPS
Inflammatory and biochemical findings	Systemic inflammation may occur but is not specific	Marked hyperferritinemia, transaminitis, cytopenias, and persistent systemic inflammation are characteristic	Inflammatory markers and organ-specific biomarkers may rise during active disease	Markedly elevated ferritin and ALT, persistent cytopenias, fever, and worsening organ dysfunction supported probable HLH; marked troponin elevation supported recurrent myocardial injury
Diagnostic framework	Involvement of at least three organs, development within one week, antiphospholipid-antibody positivity, and histopathological evidence for definite CAPS	Clinical and laboratory probability assessed using the HScore	Diagnosis based on clinical features, disease-specific investigations, and assessment of the affected organ	The patient met criteria for probable CAPS because histopathological confirmation was unavailable. An HScore of 175 corresponded to an estimated 54-70% probability of HLH
Key treatment focus	Anticoagulation, high-dose corticosteroids, plasma exchange, and treatment of the precipitating trigger	Treatment of the underlying trigger and suppression of excessive inflammation	Organ-directed immunosuppression and treatment of the underlying SLE flare	Management included anticoagulation when feasible, corticosteroids, plasma exchange, IVIG, rituximab, cyclophosphamide, and anakinra
Principal source of diagnostic uncertainty	Sepsis, SLE activity, thrombotic microangiopathy, and other causes of multi-organ failure may mimic CAPS	Sepsis, liver injury, cytopenias, and active autoimmune disease may produce similar abnormalities	Infection, ischemia, and treatment toxicity may mimic or coexist with active SLE	Persistent infection, immunosuppressive toxicity, CAPS, probable HLH, and recurrent lupus myocarditis could not always be separated with certainty

In contrast, the progression of SLE-associated APLS to CAPS is less well studied, although emerging data have begun to characterize this association. SLE flares have been proposed as a trigger for CAPS [1]. A logistic regression analysis, comparing patients with primary CAPS and those with SLE-associated CAPS, found that the latter group tended to be younger and female, with higher rates of cerebral and pancreatic involvement, and with a higher mortality rate [8]. Our patient matched this profile: she was young and female, had cerebral involvement, and ultimately passed away. In a population study of 1000 patients with APLS, eight patients developed CAPS but 75% had presented with CAPS with no prior APLS history, suggesting CAPS can be the first presentation of APLS [9]. This contrasts with our patient, who may represent a smaller subset of individuals developing CAPS despite a long-standing, well-established diagnosis of APLS.

HLH can be difficult to distinguish from other multi-system autoimmune conditions such as SLE. In a retrospective study examining 103 episodes of HLH in 98 patients with SLE, myocarditis emerged as the most frequent complication, affecting 22 patients [10]. Thus, the recurrent myocarditis in our patient may partly reflect this HLH association. Furthermore, the study also emphasized that markedly elevated levels of ALT, lactate dehydrogenase (LDH), CRP and ferritin should raise early suspicion for HLH [10]. In our patient, the initial ALT rise was suspected to be related to ischemic hepatitis. However, the combination of a high HScore and elevated ferritin ultimately suggested that the transaminitis was unlikely to be purely hepatic, and more likely secondary to HLH-related inflammation.

The co-occurrence of CAPS and HLH is exceptionally rare, with only isolated cases reported in the literature. Diagnostic overlap between the two syndromes is considerable as both can present with cytopenia, liver dysfunction, fever, and hyperferritinemia [10,11]. Although surgery and infection represent distinct precipitating insults, both may converge on overlapping downstream mechanisms involving endothelial injury, complement activation, cytokine release, and immune dysregulation [1,2]. A case report noted that preceding severe infection with malaria may have led to development of HLH in a pregnant 25-year-old woman, with concurrent pulmonary embolism and triple-positive APLS antibodies, findings consistent with a probable concomitant CAPS diagnosis [11]. Another case report described CMV infection precipitating bone marrow suppression and subsequent HLH in a CAPS patient [12]. Similarly, in our patient, HLH developed following chronic *Pseudomonas* infection and concurrent CMV reactivation, suggesting that infection may have been an important precipitating factor in subsequent HLH development. Moreover, while management strategies may overlap, distinguishing between HLH and CAPS remains critical, as the two conditions can potentiate and exacerbate one another - and failure to address concomitant disease may worsen outcomes [11].

Histopathological confirmation was not obtained for CAPS, HLH, or myocarditis. No biopsy was performed specifically to establish these diagnoses; tissue sampling undertaken during later surgical exploration for suspected necrotizing fasciitis was unrelated. The absence of tissue confirmation therefore reduced diagnostic certainty, with the postoperative presentation classified as probable CAPS, probable HLH supported by the HScore and clinical trajectory, and recurrent lupus myocarditis diagnosed through integrated clinical, biochemical, coronary, and CMR assessment.

This case therefore adds to the limited literature by demonstrating a rare sequential overlap of post-cardiac-surgery CAPS, recurrent lupus myocarditis, severe infection, and probable HLH in a patient with longstanding SLE and APLS.

Unusual timeline and refractory disease sequence

Relapsing CAPS is characterized by clinical and hematological recurrence after at least 30 days of remission, whereas refractory CAPS refers to disease that does not respond to standard triple therapy with anticoagulation, corticosteroids, and plasma exchange and/or IVIG [2]. In our patient, the initial postoperative CAPS episode demonstrated refractory features, with persistent thrombocytopenia and multisystem disease despite these therapies. The later deterioration was not considered relapsing CAPS because there was no clearly documented ≥ 30-day remission followed by a new discrete CAPS episode; instead, recurrent lupus myocarditis, severe infection, probable HLH, and progressive multi-organ dysfunction developed sequentially and overlapped with the refractory CAPS course.

Relapsing or refractory CAPS is uncommon. A case series analyzed long-term outcome data of 136 patients. The study noted 63 patients (46%) had passed away at the initial event but of the remaining 73 patients, only 11 patients had developed further APLS events, but none defined as a second CAPS event [13]. Multiple studies analyzing the CAPS registry suggest that relapsing or refractory disease is uncommon [2,3]. Additionally, antinuclear antibody positivity has been proposed as a potential risk factor for relapse [1].

Evidence describing coexistence of CAPS with recurrent lupus myocarditis is limited. Reported cases suggest that such presentations may develop postpartum, or following immunosuppressive therapy cessation, or with anticoagulation withdrawal [14]. A multicenter retrospective study comparing lupus and non-lupus myocarditis found that the lupus group had a higher frequency of conduction abnormalities (22% vs 5%), more frequent episodes of left ventricular compromise (48% vs 30%), and had higher pro-brain natriuretic peptide levels, suggesting a more severe disease course [15]. APLS was also more common in SLE patients with myocarditis than in SLE patients without myocarditis [15]. This pattern mirrors our patient’s presentation, characterized by recurrent lupus myocarditis and overlapping autoimmune syndromes.

The revised Lake Louise Criteria integrate T2-based evidence of myocardial edema with T1-based evidence of nonischemic myocardial injury in the CMR assessment of myocarditis [15]. In this patient, late gadolinium enhancement provided a T1-based finding, but the T2-based component could not be reliably assessed because T2 mapping was unavailable and the acquired T2-weighted images were nondiagnostic; T1 mapping was also unavailable. Consequently, the criteria could not be fully evaluated. Clinically apparent myocardial inflammation occurs in approximately 1-10% of patients with SLE [15]. CMR is an important noninvasive modality in the early assessment of suspected myocarditis, providing assessment of ventricular function, myocardial edema, and late gadolinium enhancement [15], but image quality and tissue characterization may be limited in critically ill patients. Where clinically feasible, repeat CMR may therefore be useful as part of longitudinal assessment of recurrent lupus myocarditis alongside clinical and biochemical markers.

Novel immunotherapy drugs

Management strategies for CAPS involve a triple therapy approach using therapeutic anticoagulation (achieving an INR > 2.0), high-dose glucocorticoids and plasma exchange [1-3]. A case series of five patients highlighted how IVIG can be an effective treatment for lupus myocarditis, leading to sustained clinical and echocardiographic remission [16]. In our patient, IVIG was similarly employed during recurrent lupus myocarditis flares, although improvement was transient.

Further management strategies for refractory or relapsing cases involve using treatment modalities with a more limited evidence base. Cyclophosphamide has been suggested to improve survival in SLE-associated CAPS [1] but worsens prognosis in primary CAPS [8]. This was similarly used in our patient’s case. Additionally, hydroxychloroquine was used as a long-term immunosuppressive agent in our patient prior to mitral valve repair. This agent may have a role in reducing platelet activation, thereby mitigating thrombotic risk [1].

Case-based and cohort data support the use of rituximab, an anti-CD20 monoclonal antibody targeting B cells, in CAPS, particularly in refractory cases [1-3]. In a cohort analysis of 22 patients receiving rituximab therapy, eight patients attained complete remission and eight patients attained partial remission, with notable reduction in ESR and anticardiolipin antibody titers [17]. CAPS involves cytokine-mediated and antibody-driven pathogenesis; thus, depletion of circulating autoantibody-producing B cells may theoretically halt progression.

Eculizumab, a terminal complement C5 inhibitor, has also shown benefit in refractory episodes of CAPS, though the evidence is limited to case reports [1-3]. Reported cases highlight its use in patients with refractory thrombocytopenia or renal involvement, with high rates of hematological and renal recovery in this subset [18]. By preventing the cleavage of C5 and subsequent formation of the membrane attack complex (MAC), eculizumab interrupts complement-mediated endothelial injury, thereby limiting thrombotic microangiopathy [18]. In this patient, eculizumab was considered as rescue therapy because of the severe refractory course and renal involvement. However, practical difficulties in securing timely access, together with rapid clinical deterioration, meant that treatment was ultimately not pursued.

Thrombopoietin receptor agonists (TPO-RAs), such as romiplostim or eltrombopag, may serve as second-line options in SLE-related ITP. In our patient, both agents were used sequentially during refractory thrombocytopenia with partial improvement in platelet counts. A large multicenter retrospective cohort study demonstrated 94% efficacy in treating SLE-associated ITP, although four arterial thrombosis events occurred, underscoring that the safety profile of TPO-RAs in lupus-associated ITP remains insufficiently characterized [19]. In contrast, a single-center study involving 12 patients demonstrated sustained platelet improvement following eltrombopag administration, even after discontinuation, without any reported thrombotic adverse events [20]. This suggests eltrombopag may contribute to improved outcomes when used cautiously in selected patients.

Multi-system collapse and neuropsychiatry element

Our patient experienced relentless multi-organ failure, including renal cortical necrosis, recurrent lupus myocarditis, cerebral infarcts, and refractory thrombocytopenia. Prolonged immunosuppression led to possible CMV reactivation, chronic *Pseudomonas* colonization, non-healing leg cellulitis, sternotomy wound breakdown, and metabolic toxicity.

Superimposed delirium and low mood were considered multifactorial rather than manifestations of a single confirmed neuropsychiatric SLE syndrome. Potential contributors included chronic corticosteroid exposure, recurrent and chronic infection, cumulative fatigue from intensive care admission, renal and metabolic disturbance, possible ongoing CAPS-related micro-thrombotic events in the cerebral vasculature, and possible lupus-associated encephalopathy.

This cumulative decline demanded daily multidisciplinary decisions across rheumatology, nephrology, hematology, cardiology, infectious diseases, intensive care, and palliative care, ultimately underscoring the devastating trajectory and human burden of refractory CAPS/HLH/APLS/SLE autoimmune overlap.

Clinical outcomes and broader implications

Outcomes vary substantially across these complications: in a cohort of SLE-associated macrophage activation syndrome, five deaths occurred among 103 episodes [10], while one myocarditis-related death occurred among 25 patients with SLE-associated myocarditis in a multicenter cohort [15]. Long-term data following CAPS also remain guarded, with 16% of initially surviving patients with available follow-up subsequently dying over a mean follow-up of 67.2 months [13].

This case advances existing knowledge by illustrating the rare sequential overlap of post-surgical CAPS, recurrent lupus myocarditis, infection, and probable HLH in a patient with longstanding SLE and APLS. Although no validated threshold for therapeutic futility can be derived from a single case, progressive multi-organ dysfunction, recurrent infection, cumulative treatment toxicity, and failure of multiple immunomodulatory therapies indicated diminishing benefit from further treatment. This case supports individualized perioperative risk assessment, counseling, and multidisciplinary planning in high-risk autoimmune patients, while important uncertainties remain regarding the relative contributions of surgery, autoimmune activation, infection, and immunosuppressive therapy to the patient’s deterioration.

Key learning points

Cardiac surgery can precipitate CAPS in predisposed autoimmune patients through a ‘double-hit’ inflammatory mechanism. The clinical boundaries between SLE, CAPS and HLH frequently overlap, creating diagnostic ambiguity and complicating management. Refractory CAPS remains rare but carries high mortality, with lupus myocarditis representing one of its more frequent manifestations. Early recognition and timely escalation to novel immunotherapies, such as rituximab or eculizumab, may improve outcomes in refractory cases. Given the cumulative toxicity of prolonged immunosuppression, treatment plans require continuous reassessment of benefit against infection risk. Early multidisciplinary team input and timely transition to palliative care are essential components of holistic, patient-centred management when therapeutic exhaustion is reached.

## Conclusions

This case exemplifies the devastating potential of overlapping autoimmune syndromes, where SLE, CAPS, HLH, lupus myocarditis, and opportunistic infection converged in a rare and relentless cascade. It highlights the diagnostic and therapeutic uncertainty that arises when immune overactivation, organ dysfunction, and treatment toxicity blur the lines between flare, infection, and failure.

Through multi-system involvement, this case demonstrates the absolute necessity of dynamic multidisciplinary team involvement, real-world use of immunomodulation, and early recognition when curative intent must transition to comfort-focused care. This case underscores the margin between immune control and immune catastrophe as well as the critical role of compassionate, team-based medicine in navigating it.
